# SINDy-Based Motion-Dependent Nonholonomic Constraint Covariance Adaptation for GNSS-Denied MEMS/INS Vehicle Navigation

**DOI:** 10.3390/mi17101187

**Published:** 2026-10-13

**Authors:** Kaiqiang Feng, Ziming Wang, Jie Li, Shengkai Shen

**Affiliations:** State Key Laboratory of Extreme Environment Optoelectronic Dynamic Measurement Technology and Instrument, North University of China, Taiyuan 030051, China

**Keywords:** MEMS IMU, GNSS-denied navigation, vehicle inertial navigation, nonholonomic constraint, pseudo-measurement covariance adaptation, invariant extended Kalman filter, sparse identification of nonlinear dynamics

## Abstract

Low-cost microelectromechanical-system inertial measurement units (MEMS IMUs) enable self-contained vehicle navigation, but their errors accumulate rapidly when global navigation satellite system (GNSS) updates are unavailable. Nonholonomic constraints (NHC) can limit this drift without external sensors, but a fixed pseudo-measurement covariance becomes inconsistent during dynamic vehicle motion. This paper presents a SINDy-inspired method for motion-dependent NHC covariance adaptation. The method preserves NHC geometry and uses completed causal IMU windows to identify a sparse relation between vehicle motion and the conditional second moment of the lateral and vertical NHC residuals. The prediction is converted into a bounded positive pseudo-measurement covariance for an invariant extended Kalman filter (InEKF). A bounded vertical covariance correction and a positive-semidefinite process-noise gate represent residual uncertainty. The learned modules output covariance quantities only, while the InEKF remains the sole estimator of navigation states. Under a strict complete-GNSS-denial protocol, the method was evaluated on three held-out vehicle sequences using route-exclusive training. Relative to fixed NHC, the 3-D position RMSE decreased from 4.135 to 1.283 m on sequence 0027 and from 14.456 to 4.293 m on sequence 0033. These reductions were 68.96% and 70.30%, respectively, and the event-level violation-induced correction score decreased by 94.41%. On the additional independent holdout sequence 0034, the proposed method reduced the 3-D position RMSE from 6.735 m with fixed NHC to 3.929 m, corresponding to a reduction of 41.67%. The results indicate that causal, motion-dependent covariance adaptation can regulate NHC information in the evaluated vehicle inertial-navigation setting.

## 1. Introduction

Reliable vehicle positioning must be maintained when global navigation satellite system (GNSS) information is degraded or unavailable. Urban canyons, tunnels, underground parking facilities, and intentional interference can interrupt the GNSS position and velocity updates on which ordinary vehicle navigation systems rely. After such a loss, the vehicle must continue with a reference-aligned dead-reckoning solution based on onboard measurements alone. This complete-GNSS-denial phase is consequential for autonomous driving, intelligent transportation, and mobile robotic platforms because attitude, velocity, and position errors can then accumulate without an external correction [1,2].

Low-cost microelectromechanical-system inertial measurement units (MEMS IMUs) make this problem both important and difficult. Their compact size, low power consumption, and low cost enable widespread vehicle deployment, but their gyroscope and accelerometer measurements contain larger stochastic noise and bias instability than those of high-grade inertial sensors. Lightweight GNSS/IRS architectures have also demonstrated that compact inertial sensors can be integrated with GNSS through Kalman filtering to improve navigation accuracy and integrity on resource-constrained UAV platforms [3]. Although that work considers GNSS-aided aerial navigation, it further motivates the development of reliable and computationally efficient inertial-fusion methods for mobile platforms. In contrast, the present study focuses on land-vehicle navigation during complete GNSS denial, when satellite-derived position and velocity updates are entirely unavailable. In a GNSS-denied MEMS/INS, these sensor errors are continuously integrated by inertial mechanization and can rapidly degrade attitude, velocity, and position accuracy. The central system-level problem is therefore to limit the navigation consequences of MEMS IMU error accumulation during satellite denial without resorting to a high-grade sensor or a continuously available external aid. Recent wheel-mounted MEMS-IMU systems illustrate the same need by combining redundant inertial measurements, wheel motion, and vehicle kinematic information to sustain autonomous positioning [4,5].

Vehicle kinematic constraints provide a practical self-contained aid for this MEMS/INS error propagation problem. In particular, the nonholonomic constraint (NHC) models the near-zero lateral and vertical velocity of a vehicle-fixed constraint point and is introduced as a pseudo-measurement in an INS filter [4,6,7]. Because it requires neither a satellite signal nor an additional exteroceptive sensor, the NHC can suppress the drift of a GNSS-denied MEMS/INS. Its geometry, however, does not imply constant reliability. Turning, side slip, suspension motion, vertical vibration, and attitude transients produce nonzero constrained velocity. A conventional fixed covariance assigns identical pseudo-measurement information to these dynamic conditions and to quiet motion. When that information is overconfident, the filter can attribute a real NHC departure to the MEMS/INS state error and impose a physically inappropriate correction.

Existing studies mitigate different aspects of this difficulty, but leave this information mismatch unresolved. MEMS calibration, redundant sensor fusion, and vehicle-model-assisted filtering improve raw inertial or auxiliary information quality [4,5,7,8,9]. Robust filtering, innovation tests, covariance matching, and Mahalanobis-distance rules reduce the effect of an inconsistent pseudo-measurement after an abnormal residual has appeared [10,11]. Learning-based approaches can bridge GNSS outages, estimate pseudo-measurement confidence, or adapt process and measurement covariances [10,12,13,14,15,16,17,18,19,20,21], but some directly regress state corrections or trajectories and many use hand-designed or innovation-driven covariance rules. These approaches do not explicitly identify, from the causal vehicle motion available to a MEMS/INS, how the expected NHC residual energy should change and how much constraint information should consequently be injected.

This paper addresses that navigation-level MEMS/INS reliability problem by modelling motion-dependent constraint information. Rather than recalibrating a MEMS IMU, replacing inertial mechanization, or directly predicting a navigation trajectory, the proposed method regulates how strongly an unchanged NHC should correct a GNSS-denied MEMS/INS under the current motion condition. The offline target is the conditional second moment of a reference body-frame NHC residual. A compact library of completed causal IMU-window descriptors is identified using the sparse-library and sequential-thresholding principle of sparse identification of nonlinear dynamics (SINDy) [22,23]. The resulting algebraic law is embedded through a bounded log-covariance mapping in an invariant extended Kalman filter (InEKF) [24]. Bounded vertical covariance matching and a positive-semidefinite process-noise gate complement the sparse prior. The learned modules output covariance quantities only, whereas the InEKF remains the sole estimator of attitude, velocity, position, biases, and extrinsics.

**Figure 1:**
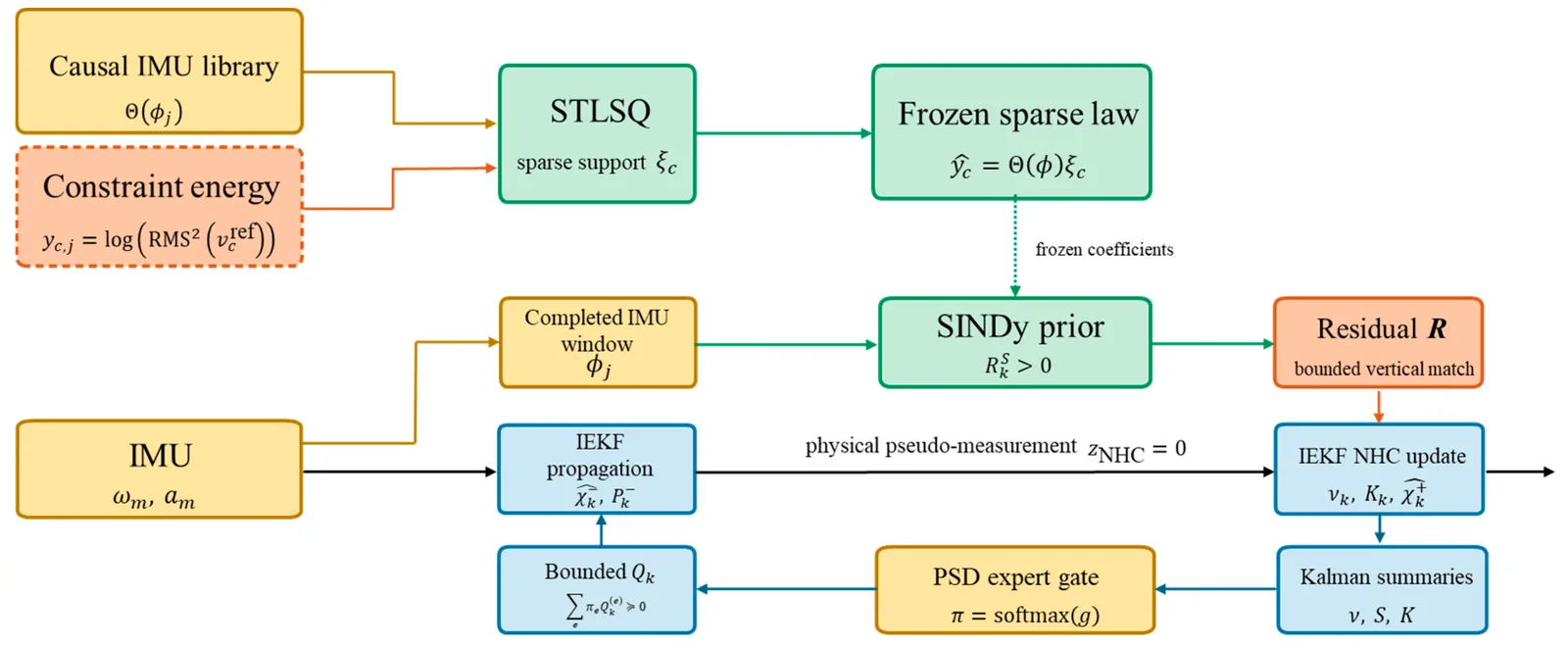
Architecture of the proposed motion-dependent NHC covariance InEKF. Reference states are used only for offline sparse-law and process-expert supervision. Online learned outputs are restricted to positive pseudo-measurement covariance and a bounded PSD process-noise covariance mixture; the InEKF alone estimates the navigation state.

The methodological contributions are threefold. First, we formulate the NHC reliability problem in a GNSS-denied MEMS/INS as a motion-conditioned residual second moment, separating fixed vehicle geometry from the information assigned to that geometry. Second, we identify an interpretable causal motion-to-information law with a sparse SINDy-inspired model instead of a black-box confidence score or direct trajectory regressor. Third, we embed the law in a bounded covariance-only InEKF interface that preserves positive covariance admissibility and retains the InEKF as the sole navigation-state estimator. Figure 1 summarizes the offline-to-online separation. The method is evaluated on three held-out vehicle routes under a strict complete-GNSS-denial protocol. After a common pre-denial initialization, every GNSS update is withheld over the complete post-denial intervals of 115.55, 165.98, and 127.53 s for sequences 0027, 0033, and 0034, respectively. The experiments test the proposed mechanism, ablate its components, and quantify causal dead-reckoning performance.

## 2. Materials and Methods

### 2.1. Estimation Setting and Motion-Dependent NHC Information

We consider the post-denial phase of vehicle navigation, beginning after GNSS updates have become unavailable. At epoch k, the online estimator may use the current and past IMU samples, the fixed vehicle nonholonomic constraint (NHC), and filter summaries computed from completed windows. It may not use a GNSS update, a reference state, a future IMU sample, or a future innovation. The objective is not to predict a navigation-state correction with a learned model. Instead, it is to determine, from the causal motion history, how much confidence the unchanged NHC should receive and how much process uncertainty should be propagated by the filter.

(1) $$X=\left(R,v,p,b_{g},b_{a},R_{ci},t_{ci}\right),R,R_{ci}\in SO(3),$$

The augmented state is defined in (1). Its navigation component, comprising rotation, velocity, and position, is represented on the extended pose group ${SE}_{2}(3)$. Gyroscope bias, accelerometer bias, and the IMU-to-constraint-frame rotation and translation are Euclidean augmented states. Accordingly, the InEKF applies a group retraction only to the navigation component and additive corrections to the auxiliary calibration states. Here and below, InEKF denotes an invariant extended Kalman filter, not an iterated EKF.

(2) $$\overset{˙}{R}=R(\omega_{m}-b_{g}-n_{g}{)}^{\wedge },$$

(3) $$\overset{˙}{v}=g+R(a_{m}-b_{a}-n_{a}),\overset{˙}{p}=v.$$

Equations (2) and (3) propagate the nominal state with measured angular rate, measured specific force, and gravity. The corresponding navigation error is represented in invariant coordinates, whereas the bias and extrinsic blocks remain Euclidean augmentations. The proposed modules do not change this inertial mechanization or generate a state estimate; they supply only the measurement and process covariance quantities used by the InEKF.

The NHC expresses the near-zero lateral and vertical velocity of a vehicle-fixed constraint point. Let $v_{c,k}$ denote this velocity in the constraint frame. The two constrained channels are selected by

(4) $$h_{c}(X_{k})=S_{c}v_{k}^{c},\;S_{c}=[\begin{array}{ccc} 0 & 1 & 0\\ 0 & 0 & 1 \end{array}].$$

The pseudo-measurement is therefore $z_{c,k}\;=\;0$. A conventional filter retains this geometry and uses the diagonal covariance and information matrix in (5). Thus, the lateral and vertical channels are adapted separately; the method does not introduce an unobserved cross-channel covariance.

(5) $$R_{c,0}=diag(R_{lat,0},R_{up,0}),\;\Lambda_{c,0}=R_{c,0}^{-1}.$$

Equation (4) is a kinematic statement about the selected velocity channels, whereas (5) determines how strongly that statement corrects the state. The geometric NHC is unchanged for the vehicle, but its statistical reliability is not necessarily unchanged. A fixed covariance assigns the same information to quiet driving and to turning, side slip, suspension motion, or vertical vibration.

For method development, the physical departure of the constraint from the reference motion is defined by

(6) $$b_{c,k}=S_{c}v_{k}^{c,ref},$$

Let $e_{c,k}$ denote the remaining pseudo-measurement error. The linearized residual is decomposed as

(7) $$\nu_{c,k}≃H_{c,k}\delta x_{k}^{-}-b_{c,k}-e_{c,k}.$$

For the residual $r_{c,k}$, the InEKF gain and Joseph covariance update are given by

(8) $$K_{k}=P_{k}^{-}H_{c,k}^{T}{\left(H_{c,k}P_{k}^{-}H_{c,k}^{T}+R_{c,k}\right)}^{-1},$$

(9) $$P_{k}^{+}=(I-K_{k}H_{c,k})P_{k}^{-}(I-K_{k}H_{c,k}{)}^{T}+K_{k}R_{c,k}K_{k}^{T}.$$

When the physical departure $b_{c,k}$ is nonzero but the filter assumes an overly small covariance, the update attributes part of that departure to navigation-state error. The resulting correction can be physically inconsistent although the NHC geometry itself is correct. The proposed method therefore adapts the information assigned to the NHC; it neither replaces the NHC equation nor regresses a position, velocity, or attitude correction.

The actual NHC residual may have a motion-dependent mean because $b_{c,k}$ need not vanish. We retain the standard zero pseudo-measurement and use a zero-mean Gaussian filtering surrogate whose effective covariance matches the conditional second moment of the total residual. This target captures both the random component and the squared motion-dependent departure, without requiring the online filter to estimate $b_{c,k}$ explicitly. Let $\varphi_{k}$ be a descriptor computed from an IMU window that has been completed at time k.

The covariance used by the zero-mean filtering surrogate is selected by conditional moment matching. Provided that the conditional residual second moment matrix is positive definite, the admissible effective covariance for the present motion condition is

(10) $$R_{c}^{*}(\phi )=E\left[q_{c}q_{c}^{T}|\phi \right].$$

For completeness, conditioning on $\varphi_{k}$ and omitting constants independent of R gives the conditional expected negative log likelihood in (11).

(11) $$L(R)=\frac{1}{2}log|R|+\frac{1}{2}tr(R^{-1}C_{\phi }).$$

Let $C_{\varphi }$ denote the matrix in (10). For every positive-definite R, the difference between (11) evaluated at R and at $C_{\varphi }$ equals twice the Kullback–Leibler divergence from $N(0,\;C_{\varphi })$ to N(0, R). It is therefore non-negative and vanishes only when $R\;=\;C_{\varphi }$. Equation (10) consequently selects the unique covariance that best represents the residual energy under the zero-mean filtering surrogate; it does not assume that the physical residual itself is conditionally zero mean.

For either scalar NHC channel c ∈ {lat, up}, the effective covariance reduces to

(12) $$R_{c}^{*}(\phi )=E[(b_{c}+e_{c}{)}^{2}|\phi ].$$

Equation (12) defines the theoretical effective-covariance target: the conditional second moment of the complete NHC residual. It contains both the motion-dependent constraint departure and the remaining random pseudo-measurement error. The sparse model introduced in Section 2.2 is trained from reference constrained velocity and therefore identifies the motion-dependent component of this target, rather than claiming to observe every contribution to the complete residual covariance. The treatment of the remaining random uncertainty is clarified after (17).

The target is useful only if increasing the effective covariance attenuates the correction caused by a violated constraint. Consider one scalar linearized channel with row vector h, $a\;=\;hPh^{T}$, and $p_{h}\;=\;Ph^{T}$. For an NHC departure b, the induced state correction is

(13) $$\delta x_{viol}(R)=\frac{p_{h}b}{a+R}.$$

For any positive-semidefinite weighting matrix W, the weighted correction energy is

(14) $$J_{W}(R)=\|\delta x_{viol}(R){\|}_{W}^{2}=\frac{b^{2}p_{h}^{T}Wp_{h}}{{\left(a+R\right)}^{2}}$$

The derivative of (14) with respect to R is given by (15). Since $a\;=\;hPh^{T}$ is non-negative, R is positive, and W is positive semidefinite, this derivative is nonpositive. Thus, for the linearized update with fixed P and h, increasing the effective covariance cannot increase the correction energy induced by the violated NHC.

(15) $$\frac{dJ_{W}}{dR}=-\frac{2b^{2}p_{h}^{T}Wp_{h}}{{\left(a+R\right)}^{3}}\leq 0.$$

This result is local because the nominal state and covariance continue to evolve in closed loop. A Euclidean norm over the complete augmented error state would also mix incompatible units. We therefore evaluate the constrained vehicle-frame velocity component over a reference-defined NHC-violation interval J. The resulting dimensionless correction score is

(16) $${VCS}_{c}(I)=\frac{\sum_{k\in I}\|E_{v,c}K_{c,k}b_{c,k}{\|}_{2}^{2}}{\sum_{k\in I}b_{c,k}^{2}}.$$

The correction score and constrained-velocity RMS provide a mechanism-level diagnostic: they test whether the covariance law actually suppresses the correction induced by a violated NHC, rather than attributing a favourable final trajectory to the covariance adaptation without examining the intervening filter action.

### 2.2. Causal Sparse Identification and Measurement-Covariance Construction

The effective information target is identified offline from labelled development data and is evaluated online without a reference state. For each completed IMU window $W_{j}$ ending at $t_{j}$, the offline scalar target for channel c is

(17) $$y_{c,j}=log\left({RMS}^{2}(v_{c}^{ref}{)}_{W_{j}}+\varepsilon \right).$$

The relationship between the theoretical target in (12) and the operational label in (17) can be made explicit by expanding the conditional second moment as

(18) $$E[(b_{c}+e_{c}{)}^{2}|\phi ]=E[b_{c}^{2}|\phi ]+2E[b_{c}e_{c}|\phi ]+E[e_{c}^{2}|\phi ].$$

When the remaining random error is conditionally zero mean and approximately uncorrelated with the motion-induced departure, the cross term is negligible. Equation (6) shows that the reference constrained velocity provides an offline estimate of $b_{c}$. Consequently,

(19) $${RMS}^{2}(v_{c}^{ref}){|}_{W_{j}}=\frac{1}{\left|W_{j}\right|}\sum_{k\in W_{j}}{\left(v_{c,k}^{ref}\right)}^{2}$$

is an empirical estimate of the motion-dependent term $E[b_{c}^{2}|\phi_{j}]$, rather than an observation of the complete second moment in (12). The constant ϵ in (17) is only a positive numerical floor that prevents the logarithm from becoming singular; it does not represent the random-error variance $E[e_{c}^{2}|\phi ]$. Reference-solution uncertainty may contribute a small amount of random energy to the label, but the complete online sensor, linearization, and filtering uncertainty is not assumed to be learned from (17). Instead, the conventional covariance $R_{c,0}$, the bounded vertical covariance correction, and the PSD process-noise gate retain and adapt the residual uncertainty not represented by the sparse motion-dependent label.

The logarithm transforms the multiplicative range of residual energy into an additive target and is consistent with the log-covariance interpolation used below. The causal descriptor is

(20) $$\phi_{j}={\left[\overset{–}{\left|\omega_{z}\right|},RMS(\omega_{x}),RMS(\omega_{y}),\overset{–}{\left|a_{y}\right|},\sigma (a_{y}),\sigma (a_{z})\right]}^{T}.$$

The library represents yaw excitation, roll and pitch motion, sustained lateral forcing, lateral transients, and vertical excitation. Every descriptor is calculated from samples no later than $t_{j}$ and standardized using development-set statistics. A nearly collinear vertical statistic is excluded, so that one physical mechanism is not represented by duplicated terms.

We then use the sparse-library and sequentially thresholded least-squares (STLSQ) principle of SINDy to fit an algebraic relation between the completed-window descriptor and log residual energy:

(21) y^c=Θ(Φ~)ξc,Θ=[1,Φ~].

At each STLSQ iteration, coefficients below the threshold are removed and the retained support is refitted:

(22) $$A_{c}^{\left(q\right)}=\{i:|\xi_{c,i}^{\left(q\right)}|\geq \lambda \},$$

(23) $$\xi_{c,A}^{\left(q+1\right)}=\Theta_{A}^{†}y_{c},\xi_{c,i}^{\left(q+1\right)}=0,i\notin A_{c}^{\left(q\right)}.$$

The intercept is retained. Sparsity is therefore part of the operational predictor, rather than a post hoc explanation of a black-box confidence score. The retained descriptor and the sign of its coefficient specify which measured motion mechanism changes the effective NHC information. Because the model maps a finite causal window to an algebraic log-energy target, it is a SINDy-inspired sparse identification model, not a differential-equation discovery model. Once fitted, the support, coefficients, and standardization statistics are fixed for online deployment.

The sparse prediction is not supplied to the filter directly. It is first converted to an admissible NHC covariance. The predicted log second moment is clipped to a finite interval around the conventional covariance:

(24) η¯c,j=clipy^c,j,logRc,0−βlog10,logRc,0+βlog10.

The bounded sparse covariance prior is then formed by

(25) $$logR_{c,j}^{S}=(1-\gamma_{c})logR_{c,0}+\gamma_{c}{\bar{\eta }}_{c,j},\;0\leq \gamma_{c}\leq 1.$$

This interface guarantees a positive covariance and recovers the fixed-NHC filter when the interpolation coefficient is zero. Quiet motion may produce a covariance below the nominal value, whereas dynamic motion can increase the covariance relative to the learned quiet level and thereby withdraw NHC information. To avoid ambiguous terminology, we report the information ratio in (26) separately from an event-to-quiet covariance ratio.

(26) $$\rho_{c,j}=R_{c,0}/R_{c,j}^{S}$$

In particular, a covariance can increase relative to quiet motion while remaining below the fixed nominal level; the two ratios should therefore not be conflated.

A window estimate cannot be used before every sample in its source window has been observed. Each sparse prediction is therefore published only at the right endpoint of its completed window and is held by zero-order hold until the next completed update:

(27) $$R_{c}^{S}(t)=R_{c,j}^{S},\;t_{j}\leq t<t_{j+1}.$$

Before the first completed window, the conventional covariance is used. This scheduling excludes centred smoothing, midpoint assignment, future interpolation, and test-reference input. Consequently, the measurement covariance available to the InEKF at time t belongs to the information set available at t.

### 2.3. Bounded Residual Uncertainty Adaptation

The sparse law is the principal motion-conditioned pseudo-measurement prior and explicitly represents the dominant lateral mechanism. A compact IMU library cannot, however, represent every vertical effect associated with road grade, suspension motion, and vehicle-body vibration. We therefore complement only the vertical channel with bounded covariance matching driven by the current and past NHC innovations. With forgetting factor ρ,

(28) C^ν,k=ϱC^ν,k−1+(1−ϱ)νkνkT,

(29) R^match,k=diag+C^ν,kHkPk−HkT.

The logarithmic discrepancy between the matched and sparse vertical covariance is clipped to a prescribed interval, and the corrected vertical covariance is then formed by (30). The correction is confidence weighted and bounded. Its lateral strength is fixed to zero, so lateral NHC information remains uniquely determined by the interpretable sparse law.

(30) $$logR_{up,k}=logR_{up,k}^{S}+\alpha_{up,k}B_{up,k}.$$

This separation prevents two adaptation modules from changing the lateral channel simultaneously: the sparse model sets lateral pseudo-measurement information, while (30) supplies only complementary vertical adaptation.

Measurement covariance and process covariance regulate different pathways in the filter. The former controls how strongly the current NHC update is trusted; the latter controls how uncertainty is carried into later propagation. From a completed trailing innovation window, we construct the state-space uncertainty statistic:

(31) C^ν,j=1Wj∑k∈WjνkνkT,Q^x,j=KjC^ν,jKjT.

The Kalman-gain mapping expresses recent innovation energy in the 21-dimensional augmented error coordinates. The online feature vector uses only completed-window summaries of lateral and vertical innovations, normalized innovation, Kalman-gain magnitude, adapted measurement covariance, and traces of (31). Reference states and future samples are excluded.

Four process-noise experts define admissible uncertainty modes for navigation motion, gyroscope bias, and accelerometer bias. For expert e,

(32) Qje=αjD(se)Q^x,jDT(se).

A compact multilayer perceptron maps these completed-window summaries to four logits. Softmax weights form the convex process-noise covariance mixture:

(33) $$\pi_{j,e}=\frac{expg_{e}(q_{j})}{\sum_{r}expg_{r}(q_{j})},Q_{j}=\sum_{e}\pi_{j,e}Q_{j}^{\left(e\right)}.$$

The construction in (33) preserves covariance admissibility for every gate output, and the Joseph measurement update in (9) preserves positive-semidefinite state covariance.

Each expert covariance is positive semidefinite by construction, while the softmax weights are non-negative and sum to one; their mixture is therefore positive semidefinite. The Joseph update is a sum of congruences of positive-semidefinite matrices and consequently retains this property.

The residual modules are deliberately restricted to admissible covariance selection. They cannot output an indefinite covariance matrix and cannot write a direct correction to rotation, velocity, position, biases, or extrinsics.

### 2.4. Causal Online InEKF Recursion

The online recursion is ordered to avoid both information leakage and an algebraic loop between an innovation and the covariance used to create it. The sparse prior is available at completed IMU-window endpoints; vertical matching uses the raw current or past NHC innovation before the final correction; and the process-noise gate is applied to subsequent propagation using completed innovation history. The complete causal update sequence is summarized in Algorithm 1.
**Algorithm 1**: Causal motion-dependent NHC covariance InEKF1. Propagate the nominal augmented state and covariance with the current IMU sample, using the process covariance published from the preceding completed history. 2. If the current IMU sample completes a sparse window, evaluate the fixed sparse law, map its output to the bounded positive NHC covariance prior, and publish that prior by zero-order hold. 3. Form the NHC innovation from the propagated state. Update the bounded vertical covariance correction using only the current and past raw innovation information, before applying the NHC correction. 4. Update the process-noise covariance from completed innovation and Kalman summaries; publish it for the subsequent propagation step rather than retroactively changing the current one. 5. Perform the NHC InEKF gain, group-state correction, additive auxiliary-state correction, and Joseph covariance update.

Only the final InEKF correction updates rotation, velocity, position, biases, and extrinsics. The sparse law, the vertical covariance correction, and the process-noise gate output covariance quantities only. This interface preserves a verifiable separation between data-driven uncertainty adaptation and physics-based state estimation.

## 3. Results

### 3.1. Experimental Setup and Complete-GNSS-Denial Protocol

All experiments are conducted by offline replay of real-world vehicle measurements from the KITTI raw dataset [2], rather than by numerical simulation. Only the complete-GNSS-denial condition is emulated; the recorded vehicle motion and IMU measurements remain unchanged. For every test run, a normally available GNSS-aided solution provides a common reference-aligned initial state at the denial epoch $t_{0}$. Beginning at $t_{0}$, all GNSS observations and GNSS-derived position or velocity updates are withheld for the remainder of the recorded sequence. The online estimator then receives only IMU samples, the fixed NHC geometry, completed causal IMU-window features, and current or past filter summaries. Reference position, velocity, and attitude are used only for offline target construction, diagnostic analysis, and performance evaluation; they are not supplied to the InEKF, the sparse covariance law, or the residual gate after $t_{0}$.

Accordingly, this is a controlled complete-GNSS-denial evaluation of post-denial dead reckoning, rather than an experiment on satellite signal acquisition, partial constellation availability, or interference detection. The available extracted dataset does not contain raw satellite observables; the evidence therefore concerns the navigation phase after all GNSS information has become unavailable. This separation makes it possible to isolate whether motion-dependent NHC information improves the inertial solution during a full denial interval.

Experiments use vehicle sequences extracted from the KITTI raw dataset [2]. The primary two-route evaluation uses 2011_09_30_drive_0027 and 2011_09_30_drive_0033, denoted 0027 and 0033. An additional independent strict-holdout evaluation is conducted on sequence 2011_09_30_drive_0034, denoted 0034. The post-denial durations are 115.55 and 165.98 s, respectively, at approximately 100 Hz. They therefore represent near-two-minute complete-denial runs rather than a common, exactly 120 s horizon. Sequence 0027 contains several turns and a localized high-turning event, whereas sequence 0033 is longer and yields substantially larger open-loop inertial drift.

To broaden the evaluation, an additional independent strict-holdout experiment was conducted on sequence 2011_09_30_drive_0034. Its post-denial interval contains 12,754 IMU samples, lasts 127.53 s, and covers approximately 922.31 m. For this experiment, sequence 0034 was excluded from all sparse-law fitting, residual-gate training, and process-expert supervision, while the remaining 40 effective sequences formed the development set. The same online configuration and complete-GNSS-denial protocol were retained without route-specific tuning.

For the primary two-route evaluation, sequences 0027 and 0033 are excluded from sparse-law fitting, residual-gate fitting, and process-expert supervision. The remaining 41 extracted road sequences form the development corpus for support-stability analysis and offline training. At t0, all navigation variants use the same reference-aligned initial attitude and velocity, representing the common pre-denial navigation solution; thereafter, their estimates evolve without test-reference input. All variants also share the same IMU mechanization, InEKF state, NHC geometry, and initial covariance.

The fixed-NHC, SINDy-R, and complete methods use one common configuration on both held-out routes, summarized in Table 1; no route-specific setting is introduced during the post-denial run. Fixed NHC retains a constant pseudo-measurement covariance. SINDy-R changes only the sparse motion-dependent NHC covariance prior. The proposed method additionally applies the bounded vertical covariance correction and the PSD process-noise gate. The 0.5 s causal window, 0.25 s update stride, STLSQ threshold of 0.4, and finite covariance bounds are held fixed across the reported comparisons.

To provide a literature-based robust-filtering comparison, the independent 0034 experiment additionally includes a Huber-robust NHC baseline, following the residual-reweighting principle commonly used in robust navigation filtering [11,25]. For NHC channel c, the current innovation $r_{c,k}$ is standardized using a robust scale $s_{c}$ fixed from the development sequences, $u_{c,k}=|r_{c,k}|/s_{c}$. With the Huber threshold δ=1.345, the weight is $w_{c,k}=1$ when $u_{c,k}\leq \delta$, and $w_{c,k}=\delta /u_{c,k}$ otherwise. The effective pseudo-measurement covariance is then inflated as ${\tilde{R}}_{c,k}=min(1/w_{c,k},100)R_{c,0}$. The lateral and vertical robust scales, 0.07155 and 0.08886 m/s, respectively, are estimated using the Gaussian-consistent median absolute deviation over the 40 development routes, with sequence 0034 excluded. This baseline uses the same InEKF state, initialization, NHC geometry, process model, and nominal covariance as Fixed NHC, and it uses neither a reference state nor a future sample online.

(34) RMSEp=1N∑k=1N‖p^k−pkref‖22.

The principal endpoint is the 3-D position RMSE accumulated over the entire post-denial interval.

### 3.2. Motion Dependence of the Reference NHC Residual

**Figure 2:**
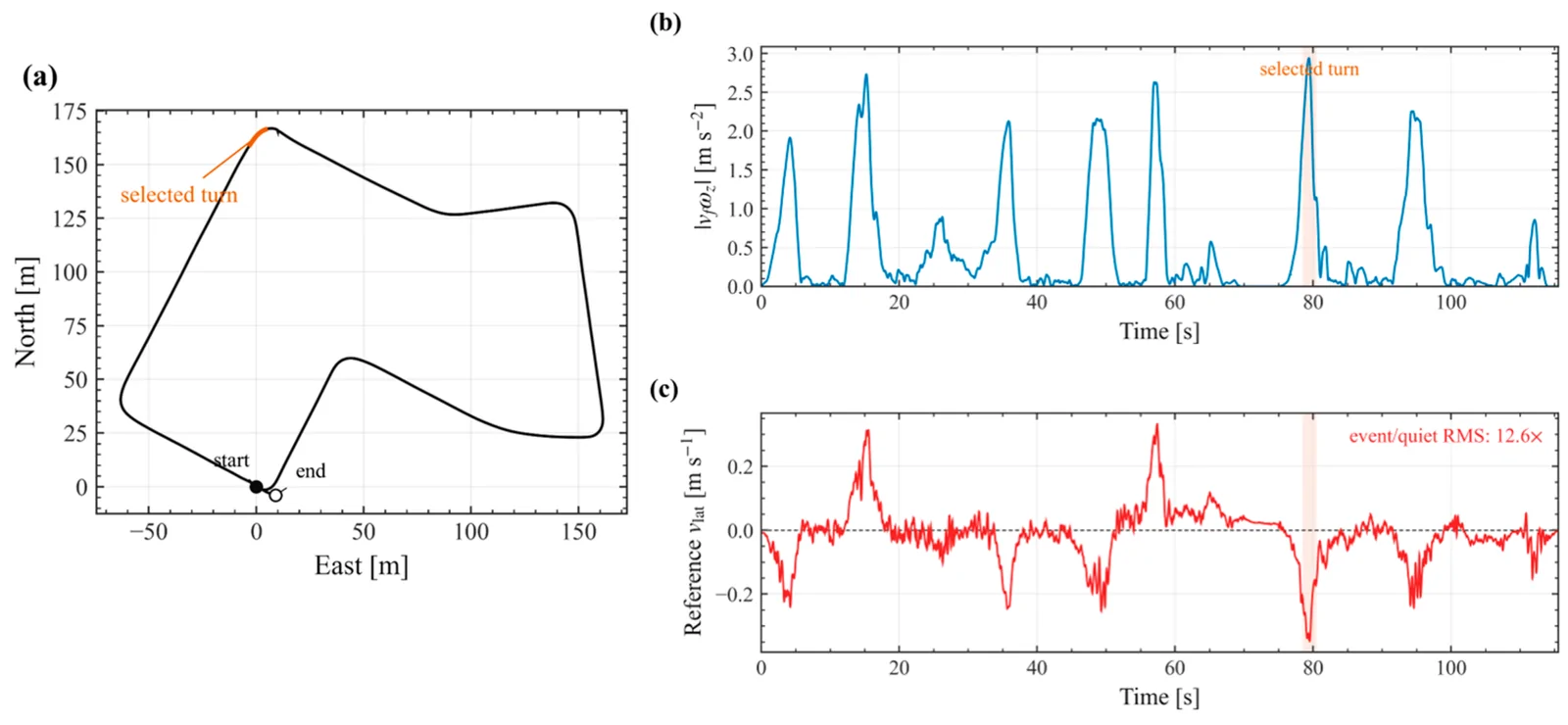
Offline reference evidence that lateral NHC validity is motion-dependent on the complete-GNSS-denial test route 0027. (**a**) Complete route and the event selected from reference turning motion. (**b**) Full-route forward-speed and yaw-rate turning excitation. (**c**) Reference lateral body-frame velocity. In the declared event, lateral-velocity RMS rises from 0.021 to 0.264 m/s; no adaptive-filter output is used to select or define the event.

The first experiment tests the physical premise before examining navigation accuracy. Figure 2 uses reference motion only as an offline diagnostic to identify a turning interval, not as an input to any online estimator. The route panel localizes the reference-defined event. The full-sequence excitation signal, defined as the nominal lateral centripetal acceleration implied by forward speed and yaw rate, rises sharply during this interval, and the reference lateral body-frame velocity departs from zero at the same time.

The quiet interval is 73.0–77.0 s and the event interval is 78.5–80.5 s. The reference lateral-velocity RMS rises from 0.021 to 0.264 m/s, a factor of 12.55, with an event peak of 0.348 m/s. The turning-excitation RMS increases by a factor of 30.81. Because the event is declared from reference motion before comparing filters, these measurements show that NHC departure is motion-dependent; they are not innovations generated by a poor post-denial estimate.

### 3.3. Causal Sparse Law on Held-Out GNSS-Denial Routes

**Figure 3:**
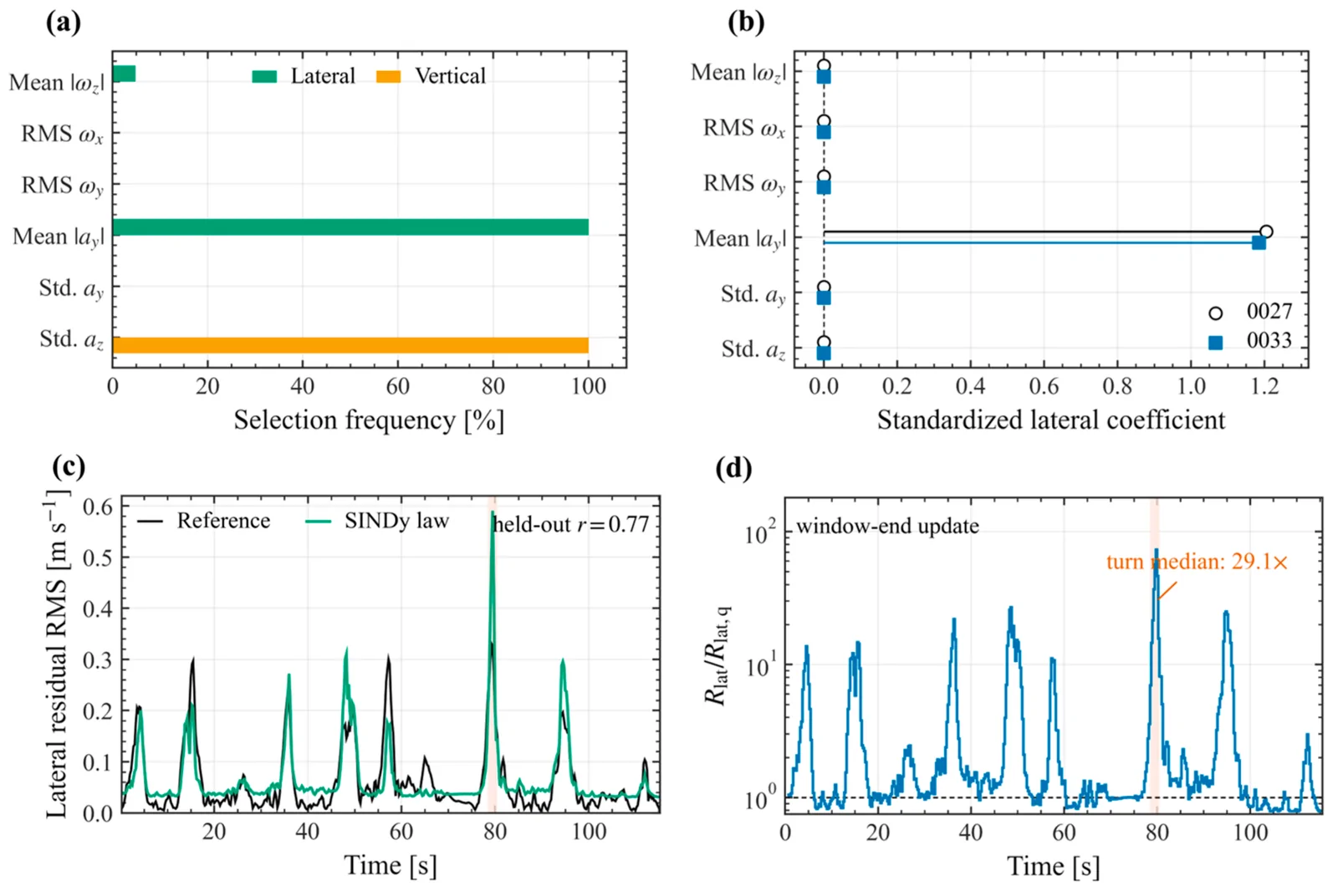
Causal sparse identification of the lateral constraint-information law under complete GNSS denial. (**a**) Selection frequency and sign consistency over development-sequence fits. (**b**) Standardized lateral coefficients for the two held-out routes. (**c**) Held-out log-energy reconstruction on 0027. (**d**) Strictly causal covariance relative to the learned quiet level. The event median is 29.10 times the quiet median; the curve is updated only at completed-window timestamps.

Figure 3 summarizes the sparse-law support, coefficient consistency, held-out reconstruction, and causal covariance schedule. The second experiment asks whether the motion-dependent constraint energy can be represented by a compact law that is available causally during denial. Across 41 leave-one-sequence-out fits in the development corpus, the lateral support selects mean absolute lateral acceleration in every fit, with a positive sign in every case. Yaw-rate magnitude is selected in only two fits, and the mean number of nonintercept lateral terms is 1.05. The vertical support likewise selects vertical-acceleration standard deviation in every fit, with a consistent positive sign and one nonintercept term.

For the two held-out test sequences, the discovered lateral laws are

(35) y^lat0027=ξ0+1.205ay–~,

(36) y^lat0033=ξ0+1.186ay–~,

where the tilde denotes standardization using development-set statistics. The positive lateral coefficient has a direct interpretation: stronger sustained lateral forcing increases the expected energy of the zero-lateral-velocity residual and should reduce the information assigned to the NHC.

On the held-out complete-denial routes, the one-term lateral law follows the dominant log-energy variation with correlations of 0.771 on 0027 and 0.760 on 0033. During the declared 0027 turn, the strictly causal lateral covariance median is 29.10 times its learned quiet-motion median. The first prediction becomes available at 0.490 s, only after the source window is complete, and every later value is updated at a completed-window timestamp and held causally until the next update.

The vertical support consistently selects vertical-acceleration variation, providing a stable physical descriptor for the complementary vertical covariance adaptation. Neither reference motion nor a future sample is used in this online covariance calculation.

### 3.4. Mechanism-Level Suppression of Violation-Induced Corrections

**Figure 4:**
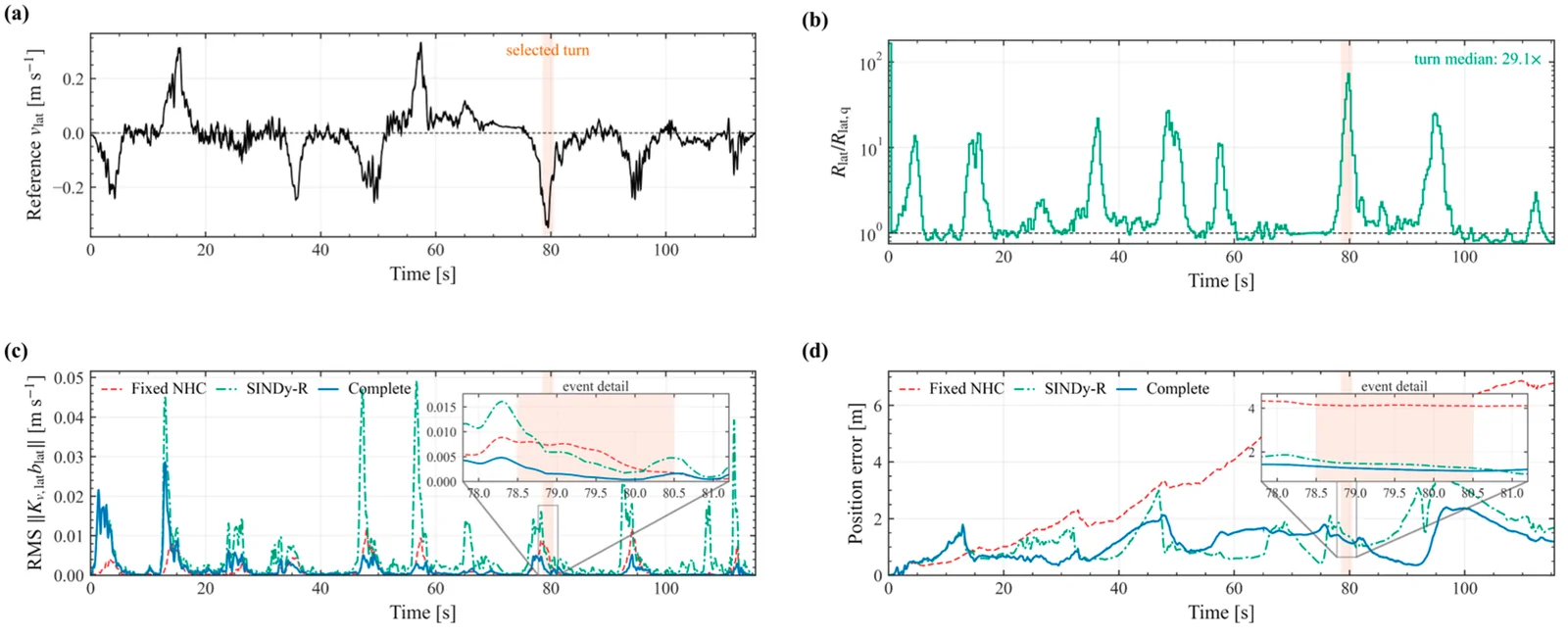
Mechanism-level reduction in violation-induced lateral-velocity corrections during the same reference-defined 0027 event. (**a**) Offline reference lateral NHC residual. (**b**) Conditional lateral covariance relative to the learned quiet level. (**c**) Offline-attributed velocity correction induced by the reference NHC departure. (**d**) Post-denial position error. The complete method reduces the event correction score by 94.41% relative to fixed NHC.

The third experiment tests the estimator mechanism rather than relying solely on final trajectory error. Figure 4 uses the same 78.5–80.5 s reference-defined interval as Figure 2 for offline attribution. The sparse covariance rises relative to its quiet-motion value only after its source window has completed, thereby reducing the lateral NHC gain through (8). The complete estimator further limits the realized correction through its bounded process-noise response. The reference residual is used only to decompose and report the correction; it is not an online filter input.

The fixed NHC produces an event violation-induced lateral correction score of 0.000477. SINDy-R reduces this score to 0.000379, a 20.57% reduction, and the complete method reduces it to 0.0000266, a 94.41% reduction. The corresponding RMS lateral velocity corrections attributable to the reference NHC departure are 0.00576, 0.00514, and 0.00136 m/s for fixed NHC, SINDy-R, and the complete estimator, respectively. Event position RMSE decreases from 4.118 to 1.418 and 1.203 m.

These results separate two roles. SINDy changes pseudo-measurement information in the physically expected direction and reduces the direct gain applied to an invalid NHC. The residual covariance modules further regulate how that local inconsistency propagates through the closed-loop error covariance. Thus, the post-denial accuracy improvement is supported by a measured correction-level mechanism rather than inferred from a favourable trajectory alone.

### 3.5. Complete-GNSS-Denial Navigation Accuracy and Ablation

**Figure 5:**
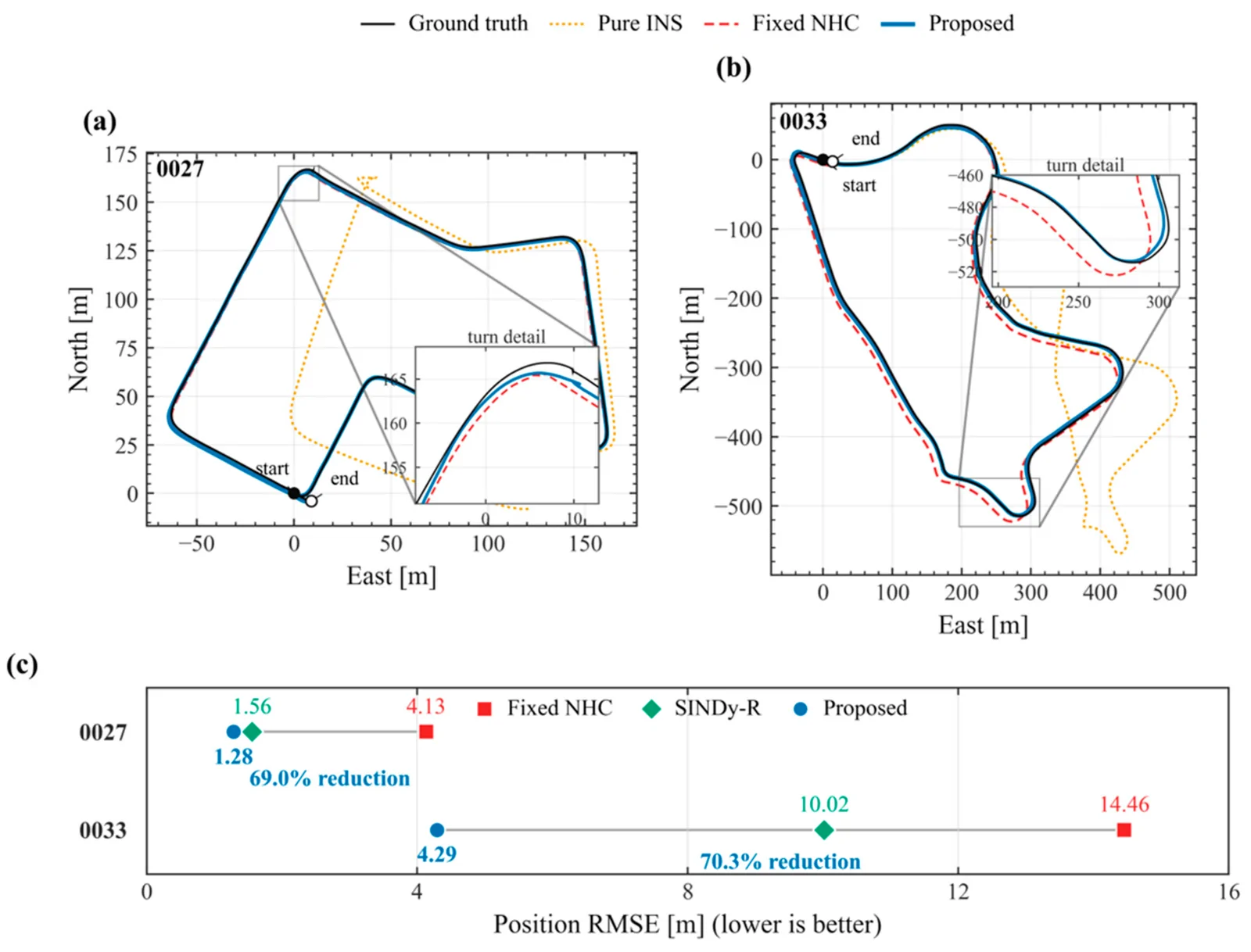
Strictly causal post-denial navigation results using one fixed configuration. (**a**) Sequence 0027. (**b**) Sequence 0033. (**c**) Component ablation in 3-D position RMSE. Pure INS is an open-loop reference; SINDy-R adapts pseudo-measurement information; the proposed method additionally applies vertical covariance correction and a bounded PSD process-noise gate. All GNSS updates are withheld after the common initialization epoch.

Figure 5 and Table 2 report the primary full-route post-denial results. Pure INS is an open-loop reference. The three constrained variants share the same initial state, IMU data, InEKF, NHC geometry, and evaluation interval; they differ only in how covariance quantities are adapted. Fixed NHC uses a constant covariance, SINDy-R applies only the sparse pseudo-measurement covariance prior, and the proposed method adds the vertical covariance correction and bounded PSD process-noise gate. No variant receives a GNSS update or a reference-state correction after the denial epoch.

On 0027, pure INS reaches 43.640 m RMSE, and fixed NHC reduces it to 4.135 m. SINDy-R reaches 1.556 m, indicating that motion-dependent measurement information supplies the principal improvement on this route. The complete method reaches 1.283 m, a 68.96% reduction relative to fixed NHC. On the longer 0033 route, pure INS reaches 204.831 m and fixed NHC reaches 14.456 m. SINDy-R reaches 10.016 m, while the complete method reaches 4.293 m, a 70.30% reduction relative to fixed NHC. These two held-out runs therefore show consistent error reduction under the same complete-denial protocol, while not establishing performance for every vehicle, route, or denial duration.

The ablation prevents the complete-method gain from being attributed to SINDy alone. On 0027, SINDy-R supplies most of the improvement and the residual covariance modules provide a smaller additional reduction. On 0033, SINDy-R improves the fixed filter but leaves substantial propagation error, and the bounded residual modules provide the larger additional gain. The same configuration is used on both routes, and neither learned branch predicts position, velocity, or trajectory.

**Figure 6:**
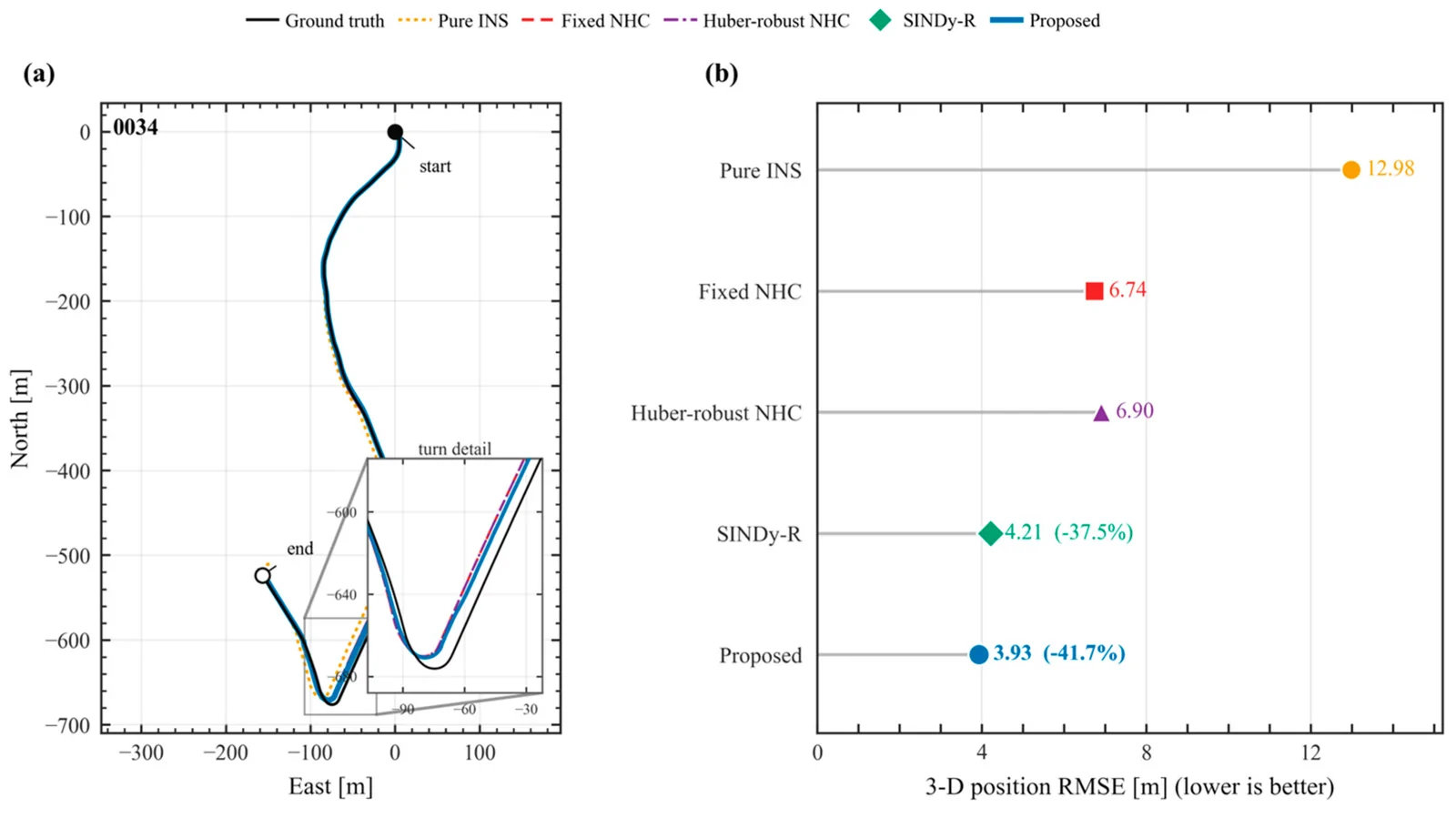
Independent strict-holdout navigation results on KITTI sequence 0034 under complete GNSS denial. (**a**) Horizontal trajectories of the ground truth, Pure INS, Fixed NHC, Huber-robust NHC, and the proposed method, with an enlarged view of the principal turning segment. (**b**) Full-interval 3-D position RMSE comparison, including SINDy-R. Sequence 0034 was excluded from all model fitting and robust-scale estimation, and no GNSS update or reference-state correction was provided after the common initialization epoch.

Figure 6 presents the additional strict-holdout results on sequence 0034. Pure INS, Fixed NHC, Huber-robust NHC, SINDy-R, and the proposed method achieved full-interval 3-D position RMSE values of 12.984, 6.735, 6.898, 4.210, and 3.929 m, respectively. The proposed method therefore reduced the RMSE by 41.67% relative to Fixed NHC, by 43.04% relative to Huber-robust NHC, and by 6.68% relative to SINDy-R. The Huber weighting was activated for 0.68% of the lateral NHC updates and 11.16% of the vertical updates, confirming that the robust mechanism did not degenerate to Fixed NHC. On this route, however, its RMSE was 2.42% higher than that of Fixed NHC. This result should not be interpreted as a general ranking of robust filters. Rather, it illustrates a possible limitation of reactive residual-based weighting in the evaluated complete-denial setting: a large innovation can arise from either a physical NHC departure or accumulated state-estimation error, and downweighting it may consequently weaken a useful constraint correction. In contrast, the proposed method predicts NHC reliability from causal vehicle-motion descriptors before the measurement update.

A formal filter-consistency evaluation based on NEES or chi-square confidence bounds is not included in the present study. Full-state NEES requires repeated independent trials and reference values for the complete 21-dimensional augmented error state, including the inertial-sensor biases and IMU-to-constraint extrinsics, which are not available in the recorded KITTI sequences. 

Although the NIS can be computed without a full reference state, its conventional chi-square interpretation assumes a correctly specified zero-mean innovation model. In the present problem, the NHC innovation can contain a deterministic, motion-dependent constraint departure and can also be temporally correlated through the adaptive covariance modules. Consequently, the reported position RMSE and violation-induced correction score demonstrate navigation accuracy and the intended correction-level mechanism, but they do not constitute a formal proof of covariance consistency. Future work will evaluate time-averaged NIS, full-state NEES, and empirical confidence coverage using controlled Monte Carlo simulations or repeated experiments with complete state-error ground truth.

### 3.6. Sensitivity, Random-Seed Stability, and Runtime

**Figure 7:**
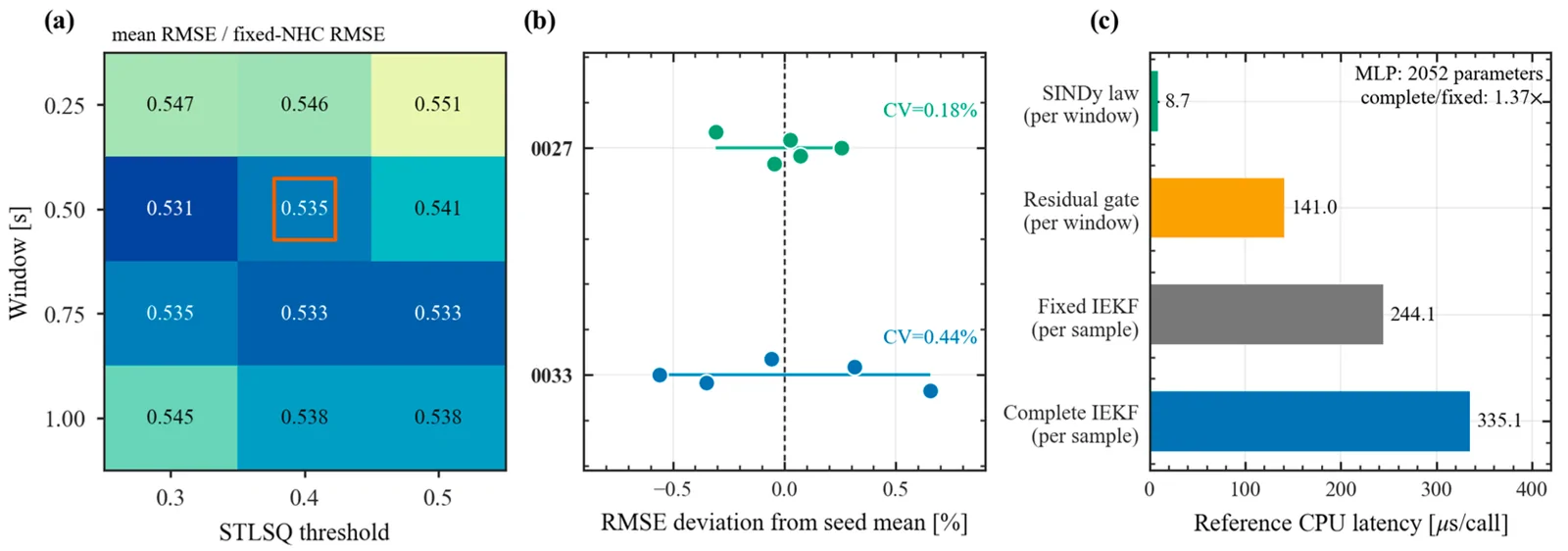
Sensitivity, seed stability, and computational cost for complete-GNSS-denial dead reckoning. (**a**) Mean normalized SINDy-R RMSE over the two held-out routes for window/threshold combinations; the 0.5 s/0.4 setting is marked. (**b**) Complete-method RMSE over five random seeds. (**c**) Reference single-thread CPU latency and filter runtime. Standalone gate latency is reported separately from the replayed causal-schedule benchmark.

Figure 7a evaluates SINDy-R over window lengths of 0.25, 0.5, 0.75, and 1.0 s and STLSQ thresholds of 0.3, 0.4, and 0.5, with all other settings fixed. Across the two held-out complete-denial routes, normalized RMSE relative to fixed NHC lies in the narrow interval 0.5305–0.5512. The 0.5 s/0.4 configuration gives normalized RMSE of 0.5346 with one lateral and one vertical nonintercept term. The analysis shows that the reported result is not confined to an isolated parameter point, while the selected setting retains a low-complexity sparse law.

Figure 7b repeats residual-gate training with seeds 0, 1, 7, 13, and 42. RMSE ranges from 1.276 to 1.283 m on 0027 and from 4.293 to 4.346 m on 0033. The corresponding coefficients of variation are 0.18% and 0.44%, indicating low seed-to-seed variability in the evaluated complete-denial setting.

The reference implementation uses Python 3.9 (Python Software Foundation, Beaverton, OR, USA), PyTorch 1.13 (PyTorch Foundation, Linux Foundation, Wilmington, DE, USA), and one CPU thread on an Intel 64 processor (Intel Corporation, Santa Clara, CA, USA). Sparse-law evaluation requires 8.72 microseconds per completed window, and the 2052-parameter MLP requires 140.96 microseconds per window. On the 11,556-sample 0027 route, median fixed-InEKF and complete-filter runtimes are 2.821 and 3.873 s, corresponding to 244.1 and 335.1 microseconds per IMU sample and a runtime ratio of 1.373. Both filter runtimes are shorter than the 115.55 s route duration, so the causal replay remains faster than real time on the stated CPU benchmark.

## 4. Conclusions

This study investigated motion-dependent NHC reliability in GNSS-denied vehicle inertial navigation, where accumulated MEMS IMU errors cannot be corrected by satellite updates. The proposed method preserves the geometric form of the NHC and models its statistical reliability through the conditional second moment of the NHC residual. A SINDy-inspired sparse law maps completed causal IMU windows to a bounded pseudo-measurement covariance. The bounded vertical covariance correction and positive-semidefinite process-noise gate account for residual uncertainty not represented by the sparse prior. The learned modules select admissible covariance quantities only, while the InEKF remains the sole navigation-state estimator.

Under a common-initialization and complete-GNSS-denial protocol, the proposed estimator reduced the 3-D position RMSE from 4.135 to 1.283 m on sequence 0027 and from 14.456 to 4.293 m on sequence 0033, relative to fixed NHC. It also reduced the event-level violation-induced correction score by 94.41%. The additional independent strict-holdout experiment on sequence 0034 further reduced the 3-D position RMSE from 6.735 m with fixed NHC to 3.929 m with the proposed method, corresponding to a reduction of 41.67%. These results support the proposed interpretation that motion-dependent covariance adaptation can withdraw NHC information when the constraint becomes less reliable during dynamic motion.

## Figures and Tables

**Table 1:** Fixed online configuration for complete GNSS denial.

Setting	Component
lateral/up NHC; fixed geometry	InEKF pseudo-measurement
$R_{lat,0}=1,$ $R_{up,0}=10$	Nominal covariance
0.5/0.25 s	Sparse window/stride
six decorrelated IMU-window features in Equation (20)	Candidate library
0.4	STLSQ threshold
β=3 decades	Covariance bound
$\gamma_{lat}=0.775,$ $\gamma_{up}=0.75$	Sparse interpolation
2 s time constant; 20 s warmup; lateral strength zero	Vertical covariance correction
24 inputs; 32-32 hidden units; four PSD experts; 2052 parameters	Process-noise gate
completed-window update; zero-order hold; trailing-only averaging	Temporal policy

**Table 2:** Post-denial navigation accuracy and ablation.

Reduction vs. Fixed	Proposed RMSE (m)	SINDy-R RMSE (m)	Fixed NHC RMSE (m)	Pure INS RMSE (m)	Sequence
68.96%	1.283	1.556	4.135	43.640	0027
70.30%	4.293	10.016	14.456	204.831	0033
41.67%	3.929	4.210	6.735	12.984	0034

## Data Availability

Publicly available data were analyzed in this study. The KITTI raw data are available at https://www.cvlibs.net/datasets/kitti/raw_data.php (accessed on 1 September 2026).
